# Development of a Patient-Centered Symptom-Reporting Application in Pharmacy Settings Using a Hierarchical Patient-Friendly Symptom List: Developmental and Usability Study

**DOI:** 10.2196/71439

**Published:** 2025-03-06

**Authors:** Seiya Watanabe, Hayato Kizaki, Satoko Hori

**Affiliations:** 1 Division of Drug Informatics Keio University Faculty of Pharmacy Tokyo Japan

**Keywords:** patient symptom monitoring, hierarchical symptom list, community pharmacy, interview survey, mobile application

## Abstract

**Background:**

Effective symptom identification, a key responsibility for community pharmacists, requires patients to describe their symptoms accurately and comprehensively. However, current practices in pharmacies may be insufficient in capturing patient-reported symptoms comprehensively, potentially affecting the quality of pharmaceutical care and patient safety.

**Objective:**

This study aimed to construct a new, hierarchical symptom list derived from the Patient-Friendly Term List of the Medical Dictionary for Regulatory Activities (MedDRA) and to develop and evaluate a mobile app incorporating this list for facilitating symptom reporting by patients in pharmacy settings. The study also aimed to assess the usability and acceptance of this app among potential users.

**Methods:**

Subjective symptom-related terms were extracted from the Patient-Friendly Term List version 23.0 of the MedDRA. These terms were systematically consolidated and organized into a hierarchical, user-friendly symptom list. A mobile app incorporating this list was developed for pharmacy settings, featuring a symptom selection interface and a free-text input field for additional symptoms. The app included an instructional video explaining the importance of symptom reporting and guidance on navigation. Usability tests and semistructured interviews were conducted with participants aged >20 years. Interview transcripts were analyzed using the Unified Theory of Acceptance and Use of Technology (UTAUT) model to evaluate factors influencing the acceptance of technology.

**Results:**

From the initial 1440 terms in the Patient-Friendly Term List, 795 relevant terms were selected and organized into 40 site-specific subcategories, which were then grouped into broader site categories (mental, head, trunk, upper limb, lower limb, physical condition, and others). These terms were further consolidated into 211 patient-friendly symptom terms, forming a hierarchical symptom list. The app’s interface design limited options to 10 items per screen to assist with decision-making. A total of 5 adults participated in the usability test. Participants found the interface intuitive and easy to use, requiring minimal effort, and provided positive feedback regarding the potential utility of the app in pharmacy settings. The UTAUT analysis identified several facilitating factors, including ease of use and the potential for enhanced pharmacist-patient communication. However, concerns were raised about usability for older adults and the need for simplified technical terminology.

**Conclusions:**

The user-friendly app with a hierarchically structured symptom list and complementary free-text entry has potential benefits for improving the accuracy and efficiency of symptom reporting in pharmacy settings. The positive user acceptance and identified areas for improvement provide a foundation for further development and implementation of this technology to enhance communication between patients and pharmacists. Future improvements should focus on addressing usability for older adults and simplifying technical terminology.

## Introduction

Patient-reported symptoms are critical indicators in health care, essential for maintaining patient safety and improving therapeutic outcomes in clinical settings [1,2]. In Japan, the Ministry of Health, Labour and Welfare launched “Pharmacy Visions for Patients” in 2015, clearly outlining the role of community pharmacies, including comprehensive monitoring of medications, as well as providing tailored drug management and guidance [3]. A key responsibility for community pharmacies in this context is to gather and monitor patient-reported symptoms, essential for identifying potential health issues and ensuring timely intervention.

For pharmacists, collecting patient-reported symptoms plays multiple critical roles in medication therapy management. First, it facilitates the early detection and monitoring of potential adverse drug reactions, enabling timely interventions to prevent serious complications. Second, comprehensive symptom information helps pharmacists assess therapeutic effectiveness and adjust medication regimens accordingly. Third, systematic symptom monitoring supports pharmacists in providing targeted patient education and improving medication adherence through a better understanding of patient experiences. These activities are fundamental to the pharmacist’s role in ensuring medication safety and optimizing therapeutic outcomes.

While health care providers traditionally collect symptom information through direct questioning, a patient-centered approach that enables patients to report symptoms at their own pace using familiar terminology may improve the accuracy and comprehensiveness of symptom reporting. Such an approach acknowledges patients as active participants in their health care, potentially leading to better identification of health-related issues and more effective interventions. A cross-sectional study demonstrated that structured symptom reporting tools can help identify numerous patient-reported symptoms and their potential associations with medications, providing valuable information for medication reviews [4]. However, another previous study showed that patients often underreported symptoms to health care providers, either because they do not attribute symptoms to the medication or do not recognize the significance of the symptoms [5].

To facilitate patient reporting, various symptom questionnaires have been developed, enabling patients to describe their experiences [6]. However, many of these tools lack thorough validation. In Japan, Nojo et al [7] introduced the “Adverse Drug Reaction Signal Check Sheet,” which lists subjective symptoms of side effects for patients taking high-risk drugs. Although limited to specific drugs, the sheet proved effective in prompting patients to communicate their symptoms during consultations with pharmacists. This approach suggests that selecting symptoms from a structured list can support patient reporting.

The Medical Dictionary for Regulatory Activities (MedDRA), a globally recognized dictionary of medical terminology, includes a Patient-Friendly Term List that reflects a wide range of symptoms reported by patients in safety databases [8]. MedDRA has a hierarchical structure with organ categories at the top, and its Patient-Friendly Term List is a supplementary list made up of the lowest-level terms of the MedDRA. This structured approach allows patients to progressively identify and report their symptoms. However, international research has indicated that patients do not fully use this list when reporting adverse drug reactions [9], suggesting challenges in the direct adoption of a symptom-reporting system.

This study aimed to construct a new hierarchical list of symptoms derived from the Patient-Friendly Term List of the MedDRA to simplify and facilitate incremental symptom selection by patients. Moreover, we aimed to develop an app incorporating this list to enhance communications between patients and pharmacists by facilitating more accurate symptom reporting.

## Methods

### Creation of the New Hierarchical, Patient-Friendly List of Symptoms

A hierarchical list of subjective symptoms was created using the Patient-Friendly Term List version 23.0 from the MedDRA. MedDRA, developed by the International Council for Harmonisation of Technical Requirements for Pharmaceuticals for Human Use, is a standardized medical terminology used in pharmaceutical regulation. The MedDRA Patient-Friendly Term List was selected for this study due to its standardized structure and global adoption in pharmacovigilance. This list consists of patient-friendly terms derived from MedDRA, ensuring that the terminology is both medically accurate and comprehensible to nonexpert users. The structured format of this list was expected to facilitate consistent symptom categorization while remaining accessible to patients, supporting pharmacists in identifying potential adverse drug reactions (ADRs) and optimizing medication therapy management.

Terms associated with subjective symptoms were extracted from the Patient-Friendly Term List and categorized by body part. Similar terms were consolidated into “symptoms,” forming the lowest level of a 3-tier hierarchical structure, with “broader site categories” and “site-specific subcategories” as the upper levels. This new hierarchical list was reviewed and refined through multiple rounds of consensus by three researchers (WS, KH, and HS). WS was an undergraduate student in the Faculty of Pharmaceutical Sciences, and KH and HS were researchers in the Faculty of Pharmaceutical Sciences.

### Development of a Patient Symptom-Reporting Application

An app was developed to incorporate the newly created hierarchical list of symptoms. The app featured a symptom selection interface, an instructional video explaining the importance of symptom reporting, and guidance on how to navigate the app. To assist with decision-making, no more than 10 options were displayed on the screen at a time. In addition to the structured symptom list, the app included a complementary free-text entry, allowing users to input symptoms that may not be represented on the list or when the listed symptoms did not fit their condition. The app is intended for use in a pharmacy setting and was designed to capture a wide range of symptoms, beyond those related to side effects of specific drugs. Apache Cordova, an open-source mobile app development framework, was used for the development. The operating system used was Android, and the app was run on a HUAWEI MediaPad T3. The content of the app was written in Japanese.

### Usability Study and Interviews

A usability study was conducted from September to October 2020 with adults aged >20 years, recruited through research flyers. Participants first completed a questionnaire on their demographics, medication use, history of ADRs, and familiarity with digital devices. They were then briefed on the purpose of the app in a pharmacy setting and the interview format. Participants tested the app on a HUAWEI MediaPad T3, entering personal and symptom data on 5 hypothetical patient scenarios (Table S1 in Multimedia Appendix 1). Following the app trial, a semistructured interview was conducted, based on the interview guide (Table S2 in Multimedia Appendix 1), to gather feedback on the app. Interviews were conducted in Japanese, focusing on impressions of the app from participants and their awareness of reporting side effects. All interviews were recorded with the consent of participants and conducted by a single researcher (WS).

### Analysis of the Interview

Thematic analysis was applied to the recorded interview transcripts using a deductive and theoretical approach. The Unified Theory of Acceptance and Use of Technology (UTAUT) model was used for this analysis. Initially proposed by Venkatesh et al [10] in 2003, the UTAUT model has been widely adopted in the medical field in recent years [11]. This model is particularly useful for understanding factors influencing the adoption of new technologies. It posits that performance expectancy (expected benefits of using new technology), effort expectancy (expectations of the ease of use and understanding of new technology), and social influence (how much the user’s decisions about technology are influenced by others) determine behavioral intention to adopt the technology. Behavioral intention, along with facilitating conditions, influence actual technology use. In addition, gender, age, experience with similar technologies, and spontaneity of use serve as general adjustment variables (moderators). The translation of these constructs was based on the work of Ono [12]. In total, 3 researchers conducted analyses to ensure objectivity.

### Ethical Considerations

On the day of the usability study and interview, the survey content, privacy protection, and plans for publishing the research results were comprehensively explained to the participants, and written informed consent to participate in the survey was obtained. The preinterview questionnaire was self-administered anonymously. Participants received compensation in the form of a gift card (Quo Card) valued at 1000 JPY for their participation in the study. This survey was conducted after being approved by the Ethics Committee for Research Involving Human Subjects, Keio University Faculty of pharmacy, following the Ethical Guidelines for Medical Research Involving Human Subjects (200213-3).

## Results

### Creation of the New Patient-Friendly Symptom List

**Figure 1:**
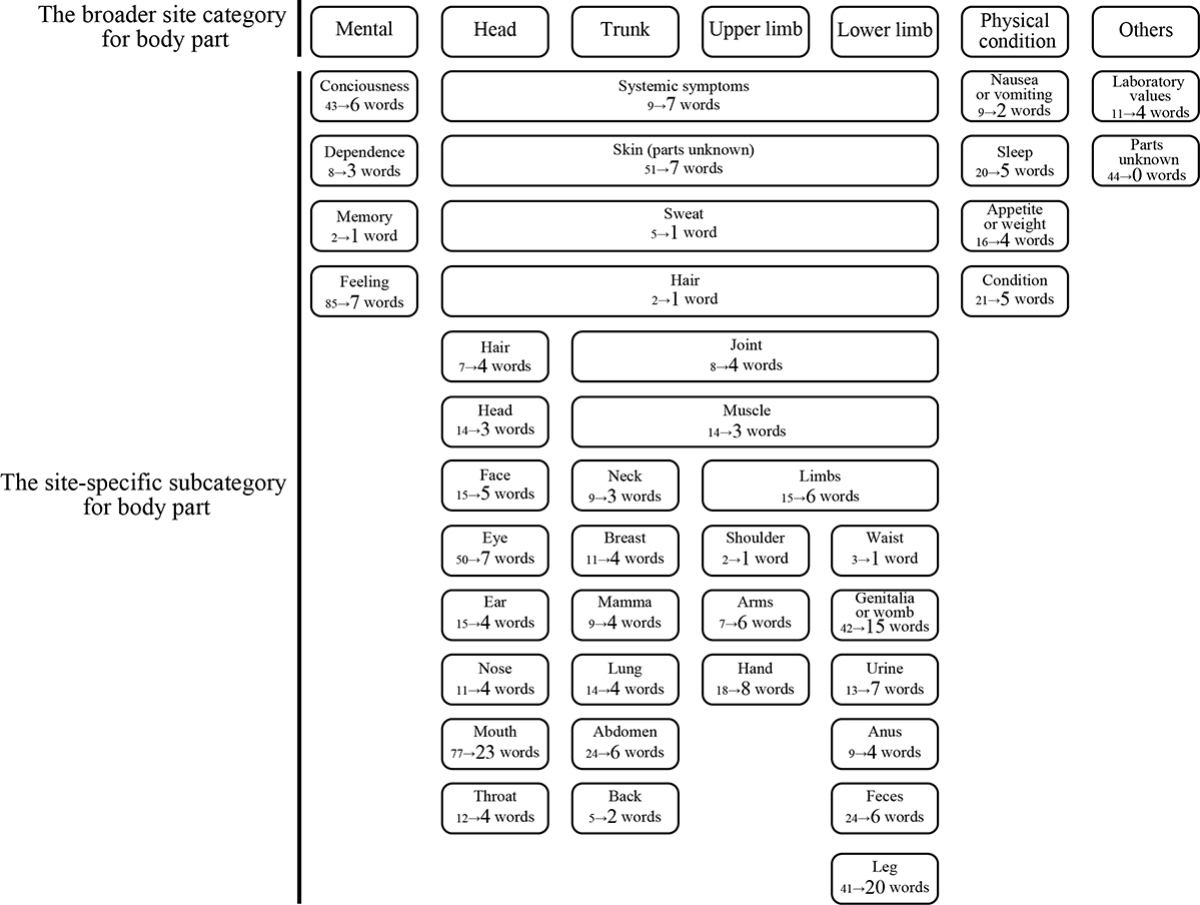
The broader site categories and site-specific categories for body parts, as well as the word counts before and after consolidation. The 795 terms were systematically sorted into 40 site-specific subcategories based on information related to the locus of impact of each word. These site-specific subcategories were grouped into 7 broad site categories: mental, head, trunk, upper limb, lower limb, physical condition, and others. Within each site-specific subcategory, similar terms were consolidated by grouping related symptoms (eg, various types of arm discomfort such as “arm paralysis” and “arm discomfort”) and creating standardized descriptive names (eg, “tingling and discomfort in the arm”) that accurately represent the consolidated symptoms. This consolidation process aimed to simplify symptom selection while maintaining clinical relevance. In this figure, the number on the left side of each arrow represents the count of terms before consolidation and the number on the right indicates the count following this unification process.

From the 1440 terms in the Patient-Friendly Term List version 23.0, duplicates with the same Japanese translations were removed, resulting in 1288 terms. Subsequently, 12 product-related terms such as “suspected counterfeit products” were excluded. In addition, 450 terms that were difficult for patients to self-identify, including those requiring a medical diagnosis or those specific to certain conditions, were removed. Another 31 terms unrelated to medications were deleted, leaving a total of 795 terms. The 795 terms were systematically sorted into 40 site-specific subcategories based on information related to the locus of impact of each word. These site-specific subcategories were grouped into 7 broad site categories: mental, head, trunk, upper limb, lower limb, physical condition, and others (Figure 1). Within each site-specific subcategory, similar terms were consolidated by grouping related symptoms (eg, various types of arm discomfort such as “arm paralysis” and “arm discomfort”) and creating standardized descriptive names (eg, “tingling and discomfort in the arm”) that accurately represent the consolidated symptoms. This consolidation process aimed to simplify symptom selection while maintaining clinical relevance, yielding 211 subjective symptoms (Figure 1). Finally, a hierarchical list of patient-friendly symptoms was created using the hierarchical structure of site categories, subcategories, and symptoms.

To integrate this list into the app, we designed a question flow that allowed for easy symptom selection. Broader and site-specific subcategories were modified and recombined into major site categories and site subcategories for an intuitive question flow (Figure S1 in Multimedia Appendix 1). As an example, the hierarchy under the major site category “upper limbs” and its subcategories is detailed in Table 1. The app included a free-text entry field labeled as “other” at each categorical level to capture symptoms that users could not find in the structured list. This feature was implemented to ensure comprehensive symptom reporting and to collect data for future improvements of the symptom list.

**Table 1:** Details of selection items (excerpt for upper limbs only). The major site categories, site subcategories, lower-level terms, and symptoms after consolidation were described in Japanese. The table presented here has been translated into English for this publication.

Major site categories and subcategories of body parts (LLT^a^)				LLT code		Symptoms after consolidation
**Upper limb**						
	**Shoulder**					
		Scapular pain	10040610		Shoulder pain	
		Shoulder pain	10040617		Shoulder pain	
	**Arm**					
		Upper limb pain	10033421		Arm pain	
		Elbow pain	10033424		Elbow pain	
		Arm rash	10037875		Arm rash	
		Arm paralysis	10003098		Tingling and discomfort in the arm	
		Arm discomfort	10049877		Tingling and discomfort in the arm	
		Arm swelling	10042680		Arm swelling and edema	
		Arm weakness	10050379		Weakness in the arm	
	**Hand**					
		Brittle nails	10006373		Abnormal nails	
		Nail discoloration	10028691		Abnormal nails	
		Finger swelling	10042694		Swelling and edema in fingers	
		Hand swelling	10042695		Swelling and edema in fingers	
		Hand rash	10019117		Hand rash	
		Cold hands	10009860		Cold sensation in hands	
		Finger pain	10033428		Hand pain	
		Hand pain	10033430		Hand pain	
		Itching of both hands	10023087		Itchy hands	
		Wrist pain	10048692		Wrist pain	
		Clumsiness	10009696		Tingling, trembling, and discomfort in fingers	
		Hand cramp	10011287		Tingling, trembling, and discomfort in fingers	
		Finger deformity	10061156		Tingling, trembling, and discomfort in fingers	
		Stiffness of fingers	10016695		Tingling, trembling, and discomfort in fingers	
		Tingling in fingers	10029837		Tingling, trembling, and discomfort in fingers	
		Tingling in hands	10049681		Tingling, trembling, and discomfort in fingers	
		Reduced dexterity	10067727		Tingling, trembling, and discomfort in fingers	
		Hand tremor	10040530		Tingling, trembling, and discomfort in fingers	

^a^LLT: lowest-level term.

### Development of a Symptom-Reporting App

**Figure 2:**
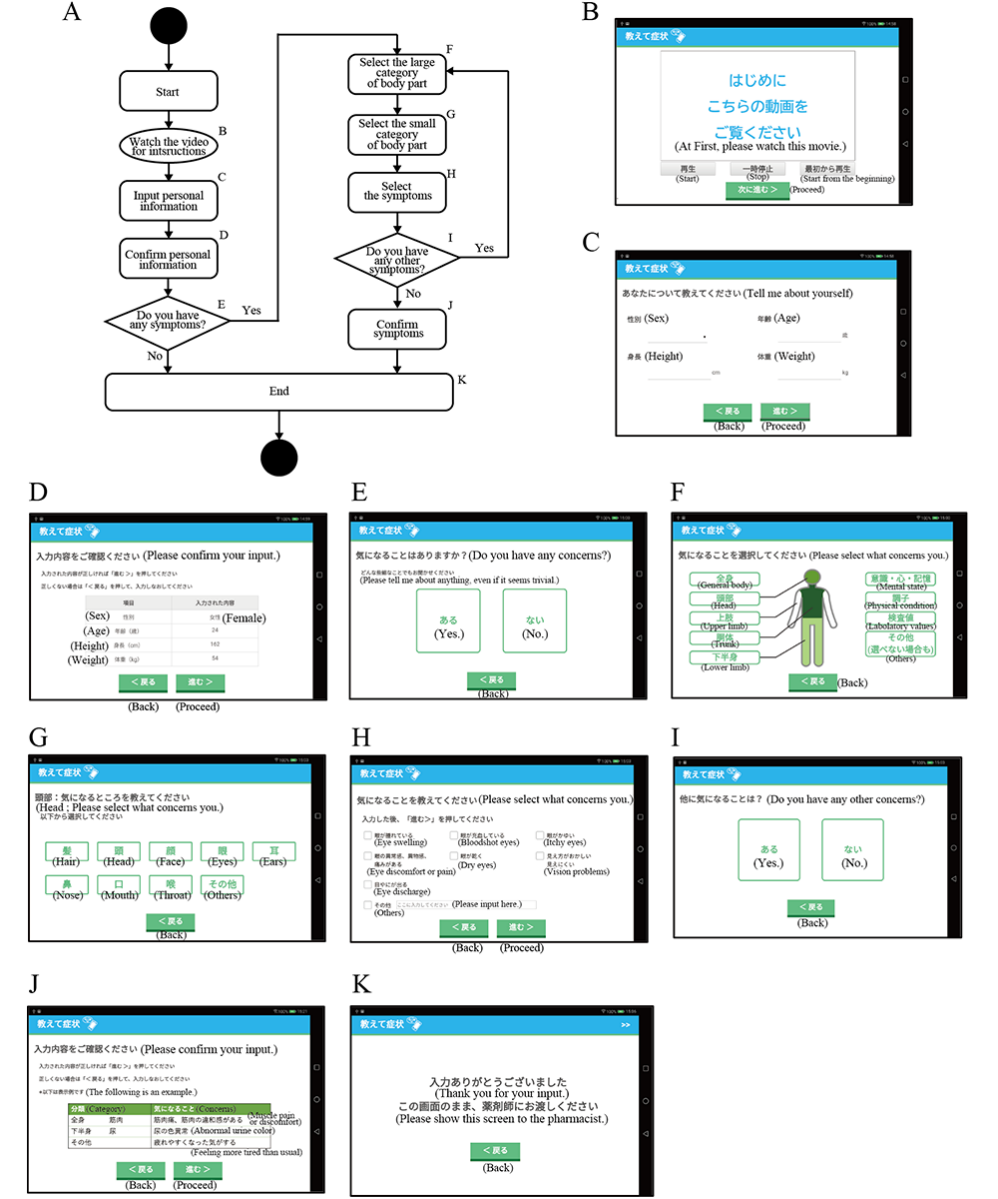
Screen transition diagram and display screen of the app. (A) Screen transition. (B) Start and video viewing section. (C) Input of personal information, (D) Confirmation of personal information. (E) Selection of the presence or absence of symptoms. (F) The large category of body parts. (G) The small category of body parts. (H) The selection of symptoms. (I) Selection of presence of other symptoms. (J) Confirmation of the selected symptoms. (K) End. The app screen in Japanese is shown since the actual app is in Japanese. English translations of the screen elements are provided in parentheses within the figure.

The screen transition diagram and display screens for the app are shown in Figure 2. Figure 2A shows the screen transition diagram of the app. The starting screen featured an instructional video explaining the purpose of the app and the importance of comprehensive symptom reporting (Figure 2B), followed by a series of intuitive selections for symptom identification (Figure 2E-J). The closed screen signaled the end of the session, prompting the user to pass the device on to the pharmacist (Figure 2K). To minimize cognitive overload, no more than 10 options were displayed per screen, making symptom selection easier and more manageable. An instructional video was included to explain the importance of symptom reporting, providing users with essential background knowledge before using the app. In addition, a free-text entry field was implemented to allow patients to describe symptoms not covered by the structured list, ensuring flexibility while maintaining structured reporting as the primary method. These features were designed to enhance the accuracy and completeness of symptom reporting, thereby improving communication between patients and pharmacists.

### Usability of the Symptom-Reporting App

In total, 5 adults participated in the usability study and interviews. Table 2 presents the demographic characteristics of the interviewees. Overall, 4 of the 5 interviewees regularly used smartphones, and 4 of the 5 interviewees had previously experienced side effects. The interviews elicited valuable opinions on the usability of the app, as summarized in Table 3, with an analysis aligned with the UTAUT model presented in Figure S2 in Multimedia Appendix 1.

**Table 2:** Basic information of interviewees.

No.	Age (years)	Sex	Occupation	Types of medicines taken, n	Experience of side effects	Smartphone or tablet	
						Possession	Frequency
1	79	Female	Unemployed	9	Yes	No	Do not use
2	24	Male	Student	3	Yes	Yes	Frequently
3	23	Male	Office worker	None	No	Yes	Frequently
4	50	Male	Sole proprietorship	3	Yes	Yes	Frequently
5	54	Female	Medical office worker	1	Yes	Yes	Frequently

**Table 3:** Opinions on app usability based on the Unified Theory of Acceptance and Use of Technology (UTAUT) model.

UTAUT category			Details
**Performance expectancy**			
	Ease of consultation	Easier to answer than to be asked Easier to tell something than in person Gives a chance to talk Unclear what the app is used for	
	Contribution for medication	May contribute to the treatment of others	
	Feedback from health care professionals	Good to have an opinion from a pharmacist Good to have continuous monitoring Good to have topics related to daily life Good to have a comfortable relationship with the pharmacist	
	Other functions	Good to provide information about medication Good to enter one’s medication status Good to be able to discuss other issues besides symptoms Good to be linked to other systems Good to be used outside the pharmacy Concerns about adding too many functions	
**Effort expectancy**			
	Ease of use	Usable without problems Easy to use selective forms Easy to be guided by selective forms Negative feelings though none of the options apply to me Difficult to enter free-text fields Difficult to check, change, or add information Difficult to use for older people who are not used to using smartphones or tablets	
	Expression of question items	Usable without problems Very detailed items Many specialized words and phrases Difficult to understand the words of the site classification Difficult to understand the way the questions are asked	
**Social influence**			
	Influence from health care professionals	Easy to answer if a health care provider asks about symptoms App that can be used by pharmacists in medication instruction	
	Information collection and management	Resistance to data being stored	
**Facilitating conditions**			
	Appearance	Easy-to-read layout Easy-to-read colors Easy-to-read font size Difficult reading text for older people	
	Device	Difficult to use keyboard No problems with the size of device Easy to use the device with horizontal screen display Probably being too small for older people	
**Behavioral intention**			
	Use of app	Want to use the app Should be used also outside of pharmacies Probably not being able to be used for older people alone Sometimes difficult to tell something even with app	

In terms of “performance expectancy,” participants expressed a preference for using the app to consult with pharmacists because “it is easier to answer than when being asked by a person,” “it is easier to communicate than doing it face-to-face,” and “it gives me a chance to talk to someone.” Some participants suggested that “it would be beneficial to receive expert opinions as feedback from a pharmacist.” In terms of “effort expectancy” (expectations about the ease of use and understanding of new technology), usability and expressions were both deemed “usable without problems,” while issues such as “too many technical terms” and “I feel bad when there are no applicable choices” were identified. Regarding “social influence” (the extent to which the user’s technology decisions are influenced by others), opinions included “it is easy to answer when a medical professional asks about symptoms” and “pharmacists can use it in medication guidance,” indicating that the influence of medical professionals contributes to the behavioral intention to use the technology. Regarding the “facilitating conditions,” it was suggested that there should be no problems with the design and terminal. Based on the above, all participants were positive about their behavioral intention to use the app, and some suggested that the app should be used outside pharmacies. Concerns were raised regarding the use of the app by older adults.

Several factors that did not align with the UTAUT model but significantly affected how ADRs reported to health care providers were identified. These factors included “I recognize pharmacist as a person to consult, but do not want to consult,” “I will not consult if not sure of side effect,” “I will not consult if the side effect is minor or not necessary to discuss by myself,” and “I will not consult if I think it is a side effect.” In addition, factors specifically affecting the reporting of ADRs to health care providers comprised “I recognize that it is an ADR but do not want to discuss it with a pharmacist,” “I do not discuss it unless I am sure it is an adverse drug reaction,” “I do not discuss it unless I am sure it is an adverse drug reaction,” and “I feel uncomfortable reporting an adverse drug reaction.”

## Discussion

### Principal Findings

In this study, we developed a hierarchical list of symptoms based on the MedDRA Patient-Friendly Term List and created an app that enables patients to easily select symptoms. The potential use of the app in pharmacies was well received, indicating that it could significantly aid patients in communicating their symptoms more effectively.

Our newly created list comprised 211 symptoms, and to improve user-friendliness, we implemented a “questionnaire flow” within the app, based on the hierarchical structure. This approach allows users to systematically select symptoms by navigating through major site categories, site subcategories, and detailed symptoms. Participants using the app in 5 hypothetical patient scenarios praised its ease of use, confirming the feasibility of using the hierarchical list of symptoms and the app for effective symptom selection. Previous research revealed that approximately 20% of reports on side effects used patient-friendly terms, with the majority opting for free-text entries [5]. By limiting displayed options to 10 or fewer, while providing a free-text input field for unlisted symptoms, our app may improve the ease of symptom identification. This design feature was further supported by the overall positive feedback from the participants. Future updates to the symptom list, informed by the analysis of real-world, free-text entries, could further streamline symptom reporting.

Participants in our study expressed their opinions about reporting usual self-aware symptoms such as “I do not discuss subjective symptoms unless I am sure they are side effects” and “I do not mention side effects if they seem minor or unnecessary to report,” which aligns with reasons reported in previous research for underreporting symptoms to health care providers [5]. These responses highlight common barriers to symptom reporting in health care settings, including patients’ uncertainty about whether symptoms are drug-related and their hesitation to mention symptoms they perceive as minor. To address these universal challenges, our app was designed with a structured interface and a hierarchical organization of symptoms, guiding patients through a systematic symptom-reporting process. The hierarchical structure of the app enables patients to progressively identify and select symptoms, potentially increasing the comprehensiveness of reported symptoms while reducing psychological barriers to reporting even minor or uncertain symptoms. Positive feedback from participants, such as “easier to answer than being asked by someone,” “easier to communicate than doing it face-to-face,” and “becomes a trigger to talk,” suggests that this structured approach could effectively address these reporting barriers. In addition, participants’ feedback indicating the app’s effortlessness (“can use without a problem”) and willingness to use (“I want to use it”) suggests its potential for successful implementation in pharmacy practice.

From the perspective of health care providers, pharmacists can review these structured patient reports before medicine consultation, enabling more focused and efficient discussions. These features, combined with the ability of the app to document patient-reported outcomes systematically, provide a foundation for continuous monitoring of therapy effectiveness and early detection of drug-related problems.

While our usability study received positive feedback regarding the potential of the app, we also identified several usability issues, particularly among older users. A participant aged 79 years expressed concerns, stating “I might not be able to use it on my own,” and other feedback pointed to specific issues such as “The size may be too small for elderly users” and “The text might be difficult to read.” Given that many customers of pharmacies are older adults, future usability assessments should focus on this population.

Implementing our app in pharmacy settings strategically aligns with the evolving health care landscape in Japan, particularly in aging societies where pharmacies are expanding beyond traditional medication dispensing [3,13]. Pharmacies offer several unique advantages as ideal implementation sites, including their high accessibility, regular patient visits, and prescription preparation waiting time, which offers a natural opportunity for symptom reporting without disrupting workflow. While the app could be valuable in various health care settings, including clinics and hospitals, pharmacies offer unique advantages. Pharmacists’ expertise in medication, combined with their frequent patient interactions, makes them ideal providers for continuous symptom monitoring and self-care support. Their role has evolved from traditional medication dispensing and adverse effect monitoring to more comprehensive patient care support. Integrating the app into pharmacy practice could particularly support patient engagement in their own health care by enabling systematic symptom reporting in a familiar, low-pressure environment. This approach not only supports pharmacists’ evolving role in comprehensive patient care but also promotes active patient participation in medication management.

Future research should systematically evaluate the effectiveness of the app in achieving its intended outcomes. Key areas of investigation include the impact of the app on the number and types of symptoms reported. Quantitative measurements will be valuable in assessing the impact on pharmaceutical interventions, including the identification of drug-related problems and subsequent care recommendations. Further research opportunities lie in evaluating improvements in the quality of patient-pharmacist communication and the effect of the app on the efficiency of pharmacy workflow. These investigations would benefit from including a larger, more diverse patient population in real-world pharmacy settings, with particular attention to older adults who represent a significant portion of pharmacy patients.

Although our sample size of 5 participants is consistent with recommendations for initial usability testing suggesting that 5 users can identify approximately 80% of major usability issues [14], this small sample size limits the generalizability of our findings. This limitation highlights the need for additional testing with a larger and more diverse user group to ensure the app meets the needs of all potential users in pharmacy settings.

### Conclusions

We successfully developed a new hierarchical list of symptoms and an accompanying app. The app, designed to enable users to select symptoms from a structured list, was positively accepted and showed strong potential for improving patient behavior in symptom reporting. Future improvements to the design of the app design, particularly for older users, will further enhance its utility in both pharmacy settings and broader health care environments.

## Appendix

Multimedia Appendix 1 Supplementary materials including hierarchical structure of symptom selection categories, UTAUT model application results, model patient profiles, and interview guide. UTAUT: Unified Theory of Acceptance and Use of Technology.

## Abbreviations

ADR: adverse drug reaction
MedDRA: Medical Dictionary for Regulatory Activities
UTAUT: Unified Theory of Acceptance and Use of Technology

## Data Availability

The datasets generated or analyzed during this study are not publicly available due to privacy or ethical restrictions but are available from the corresponding author on reasonable request.
