# Supplementary material for: Development of a Patient-Centered Symptom-Reporting Application in Pharmacy Settings Using a Hierarchical Patient-Friendly Symptom List: Developmental and Usability Study
Source: JMIR Hum Factors. 2025 Mar 6;12:e71439. doi: 10.2196/71439 (PMC11926441; doi:10.2196/71439)
Supplement: Multimedia Appendix 1 [file humanfactors_v12i1e71439_app1.docx]

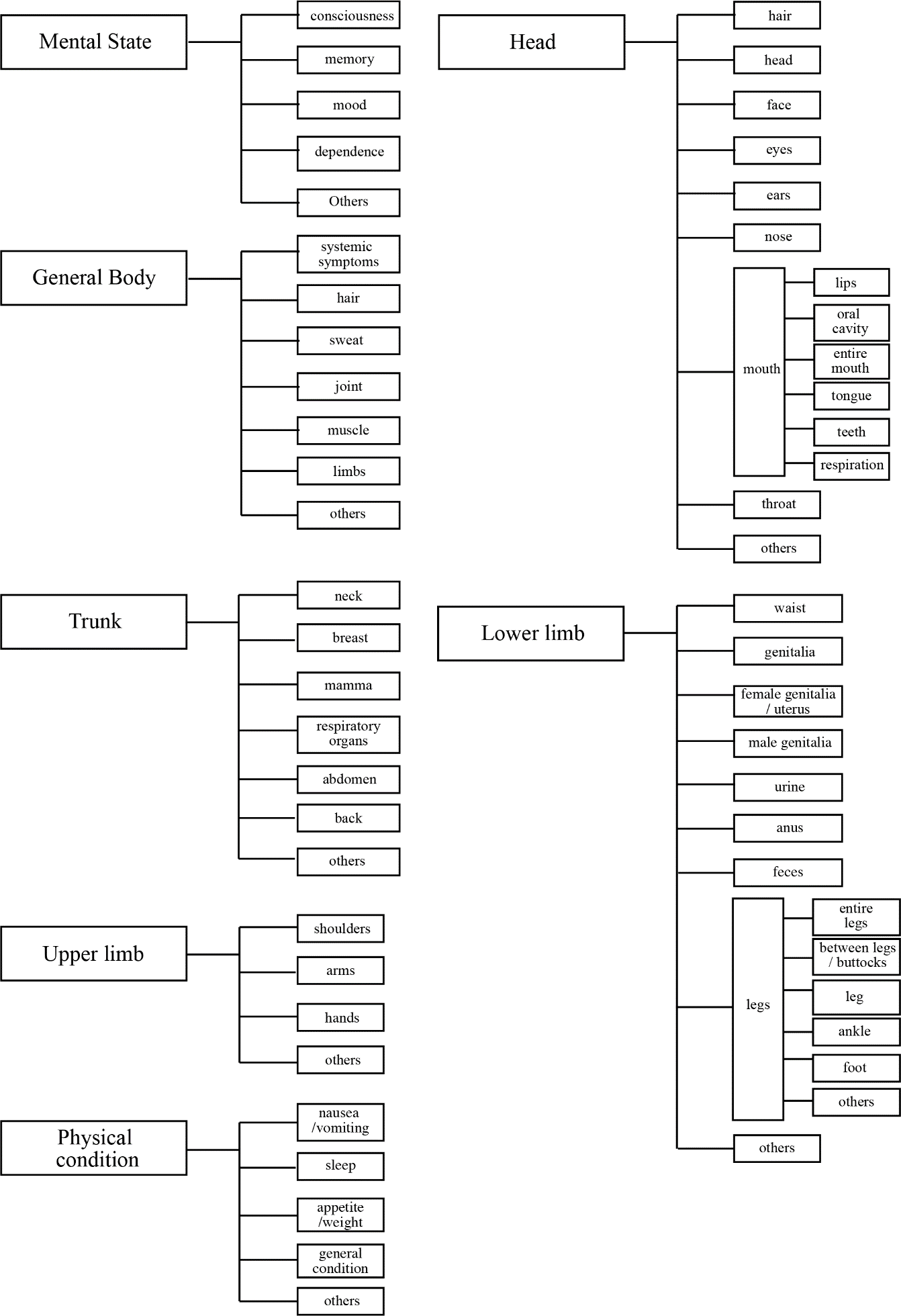


**Figure S1. Hierarchical structure of selection items (major site categories and site subcategories of body parts)**

The hierarchical structure of the major site categories and subcategories is shown in Japanese (the selection items in the actual application are described in Japanese). Under site subcategories, 211 words describing symptoms were selected. Other items were provided under all major site categories and entered in the form of free descriptions.


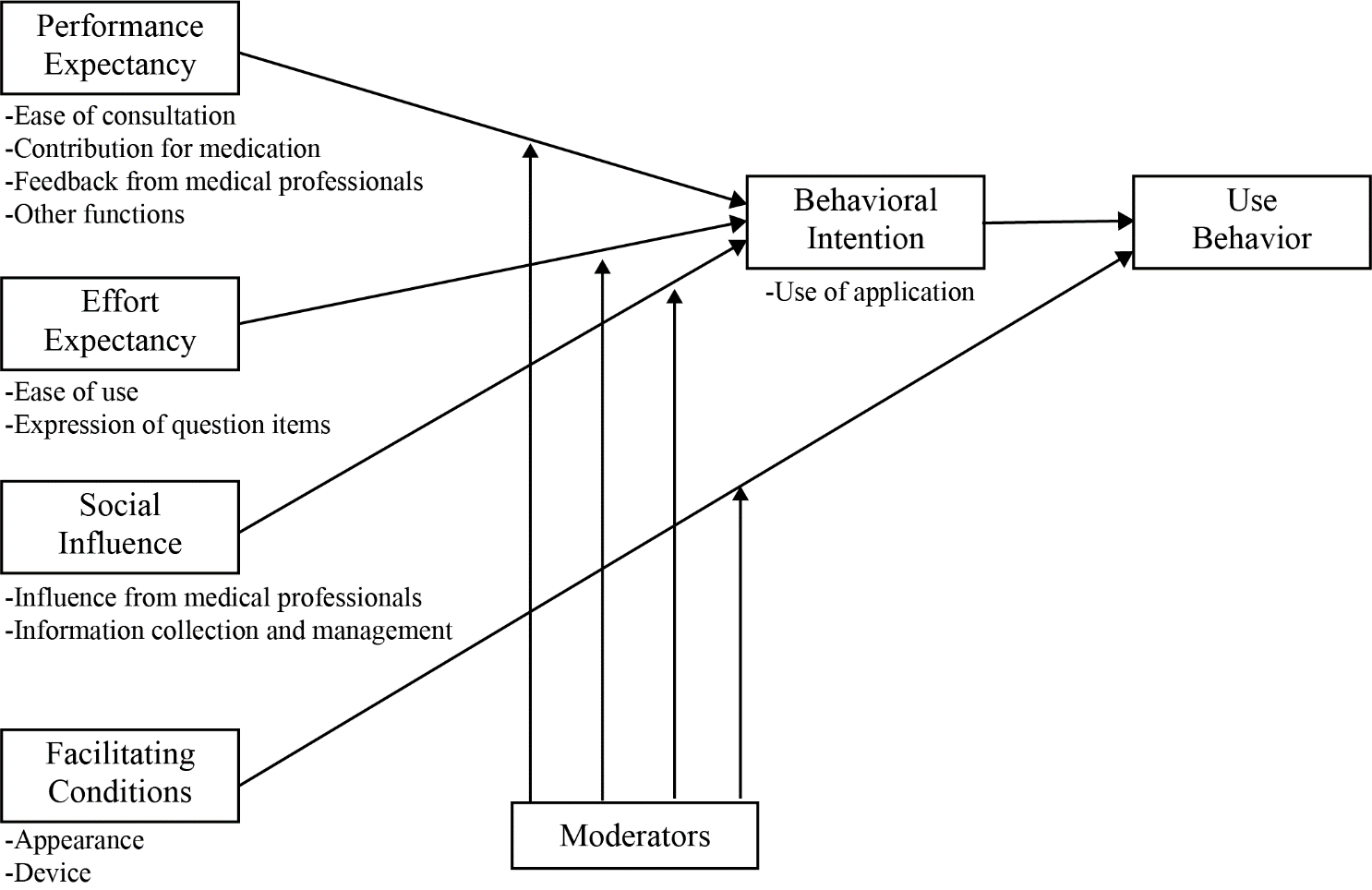


**Figure S2. Fitting research results to the Unified Theory of Acceptance and Use of Technology (UTAUT) model**

Categories extracted from the analysis of the interview survey were identified by applying the UTAUT model. In the analysis, the Japanese version of the UTAUT model was used for fitting.

Table S1. Model patients

| No. | Basic Information | Episode |
| --- | --- | --- |
| 1 | Female  37 years old  162 cm  54 kg | I went to see a doctor two weeks ago and was diagnosed with dyslipidemia and prescribed medication. I have muscle aches and pains in my arms and legs, even though I have not been exercising recently. It may be my imagination, but my pee seems to be red. |
| 2 | Male  43 years old  181cm  80 kg | A week ago, I was injured and went to an orthopedic surgeon. I was offered a compress and painkillers. Lately, I feel tired easily, probably due to my busy work. I have no appetite. |
| 3 | Female  58 years old  148 cm  44 kg | I have epilepsy and have been taking medication for a long time. When I went to my doctor two weeks ago, a new medication was prescribed. Lately, I feel like I have red spots on my body. I also have a feverish feeling. |
| 4 | Male  65 years old  173 cm  65 kg | I am diabetic and have been taking diabetic medications for a long time. Three days ago, I visited an ear, nose, and throat (ENT) specialist for a fever and was prescribed several medications, including an antibiotic. The fever went down, but I began to have cold sweats and a feeling of weakness. |
| 5 | Female  72 years old  154 cm  47 kg | I recently started taking a new medication for the control of blood pressure and my blood pressure has decreased a little. I sometimes feel dizzy due to anemia. As I recall, I think the food tasted strange lately, probably because I have not been feeling well. |

Table S2. Interview guide

| step | | Details |
| --- | --- | --- |
| Introduction | Explanation of the aims and precautions of the interview | ■ Explanation of the interview aims ■ Explanation of interview precautions |
| Main topic | About application | ■ Appearance What do you think about the appearance of the application? |
|  |  | ■ Usability  What do you think about the usability of the application? |
|  |  | ■ Representation What do you think about the representation in the application? |
|  |  | ■ Other functions  Are there any other features or information you wish you had? |
|  | About the adverse drug reaction (ADR) reports | ■ Motivation  When do you report your ADR?  What makes you report your ADR? |
|  |  | ■ Advantage of using the application  Do you think you are motivated to report your symptoms if you use this application? What feedback would you like to receive on the information you have entered to the application?  Are there any concerns about entering symptoms to application? |
|  | For the future | ■ When it becomes available as an application, do you want to use this application? What do you think about the use of this application in pharmacies? |
| Closing | closing | ■ Confirmation of other questions Do you have any other questions or suggestions about the application? |
